# Similar Limited Protection Against Severe Acute Respiratory Syndrome Coronavirus 2 Omicron Infection in Vaccinated Individuals With HIV and Comparable Controls

**DOI:** 10.1093/ofid/ofae380

**Published:** 2024-07-08

**Authors:** Myrthe L Verburgh, Anders Boyd, Maarten F Schim van der Loeff, Margreet Bakker, Ferdinand W N M Wit, Marc van der Valk, Marloes Grobben, Lisa van Pul, Khadija Tejjani, Jacqueline van Rijswijk, Marit J van Gils, Neeltje A Kootstra, Lia van der Hoek, Peter Reiss, AGEhIV Cohort Study, P Reiss, F W N M Wit, M van der Valk, A Boyd, M L Verburgh, I A J van der Wulp, M C Vanbellinghen, C J van Eeden, M F Schim van der Loeff, J C D Koole, L del Grande, I Agard, S Zaheri, M M J Hillebregt, Y M C Ruijs, D P Benschop, A el Berkaoui, A Boyd, F W N M Wit, N A Kootstra, A M Harskamp-Holwerda, I Maurer, M M Mangas Ruiz, B D N Boeser-Nunnink, O S Starozhitskaya, L van der Hoek, M Bakker, M J van Gils, L Dol, G Rongen, S E Geerlings, A Goorhuis, J W R Hovius, F J B Nellen, J M Prins, T van der Poll, M van der Valk, W J Wiersinga, M van Vugt, G de Bree, B A Lemkes, V Spoorenberg, F W N M Wit, J van Eden, F J J Pijnappel, A Weijsenfeld, S Smalhout, I J Hylkema - van den Bout, C Bruins, M E Spelbrink, P G Postema, P H L T Bisschop, E Dekker, N van der Velde, R Franssen, J M R Willemsen, L Vogt, P Portegies, G J Geurtsen, I Visser, A Schadé, P T Nieuwkerk, R P van Steenwijk, R E Jonkers, C B L M Majoie, M W A Caan, B J H van den Born, E S G Stroes, S van Oorspronk

**Affiliations:** Amsterdam University Medical Centers, University of Amsterdam, Infectious Diseases, Amsterdam, The Netherlands; Amsterdam Institute for Infection and Immunity, Infectious Diseases, Amsterdam, The Netherlands; Amsterdam University Medical Centers, University of Amsterdam, Department of Global Health, Amsterdam Institute for Global Health and Development, Amsterdam, The Netherlands; Amsterdam University Medical Centers, University of Amsterdam, Infectious Diseases, Amsterdam, The Netherlands; Amsterdam Institute for Infection and Immunity, Infectious Diseases, Amsterdam, The Netherlands; HIV Monitoring Foundation, Amsterdam, the Netherlands; Public Health Service of Amsterdam, Infectious Diseases, Amsterdam, The Netherlands; Amsterdam University Medical Centers, University of Amsterdam, Infectious Diseases, Amsterdam, The Netherlands; Amsterdam Institute for Infection and Immunity, Infectious Diseases, Amsterdam, The Netherlands; Public Health Service of Amsterdam, Infectious Diseases, Amsterdam, The Netherlands; Amsterdam Institute for Infection and Immunity, Infectious Diseases, Amsterdam, The Netherlands; Amsterdam University Medical Centers, University of Amsterdam, Medical Microbiology and Infection Prevention, Laboratory of Experimental Virology, Amsterdam, The Netherlands; Amsterdam University Medical Centers, University of Amsterdam, Infectious Diseases, Amsterdam, The Netherlands; Amsterdam Institute for Infection and Immunity, Infectious Diseases, Amsterdam, The Netherlands; HIV Monitoring Foundation, Amsterdam, the Netherlands; Amsterdam University Medical Centers, University of Amsterdam, Infectious Diseases, Amsterdam, The Netherlands; Amsterdam Institute for Infection and Immunity, Infectious Diseases, Amsterdam, The Netherlands; HIV Monitoring Foundation, Amsterdam, the Netherlands; Amsterdam Institute for Infection and Immunity, Infectious Diseases, Amsterdam, The Netherlands; Amsterdam University Medical Centers, University of Amsterdam, Medical Microbiology and Infection Prevention, Laboratory of Experimental Virology, Amsterdam, The Netherlands; Amsterdam Institute for Infection and Immunity, Infectious Diseases, Amsterdam, The Netherlands; Amsterdam University Medical Centers, University of Amsterdam, Experimental Immunology, Amsterdam, The Netherlands; Amsterdam Institute for Infection and Immunity, Infectious Diseases, Amsterdam, The Netherlands; Amsterdam University Medical Centers, University of Amsterdam, Medical Microbiology and Infection Prevention, Laboratory of Experimental Virology, Amsterdam, The Netherlands; Amsterdam Institute for Infection and Immunity, Infectious Diseases, Amsterdam, The Netherlands; Amsterdam University Medical Centers, University of Amsterdam, Medical Microbiology and Infection Prevention, Laboratory of Experimental Virology, Amsterdam, The Netherlands; Amsterdam Institute for Infection and Immunity, Infectious Diseases, Amsterdam, The Netherlands; Amsterdam University Medical Centers, University of Amsterdam, Medical Microbiology and Infection Prevention, Laboratory of Experimental Virology, Amsterdam, The Netherlands; Amsterdam Institute for Infection and Immunity, Infectious Diseases, Amsterdam, The Netherlands; Amsterdam University Medical Centers, University of Amsterdam, Experimental Immunology, Amsterdam, The Netherlands; Amsterdam Institute for Infection and Immunity, Infectious Diseases, Amsterdam, The Netherlands; Amsterdam University Medical Centers, University of Amsterdam, Medical Microbiology and Infection Prevention, Laboratory of Experimental Virology, Amsterdam, The Netherlands; Amsterdam University Medical Centers, University of Amsterdam, Infectious Diseases, Amsterdam, The Netherlands; Amsterdam Institute for Infection and Immunity, Infectious Diseases, Amsterdam, The Netherlands; Amsterdam University Medical Centers, University of Amsterdam, Department of Global Health, Amsterdam Institute for Global Health and Development, Amsterdam, The Netherlands; Amsterdam University Medical Centers, University of Amsterdam, Global Health, Amsterdam, The Netherlands

**Keywords:** HIV, incidence, Omicron variant, SARS-CoV-2, serology

## Abstract

**Background:**

Little is known about the risk of severe acute respiratory syndrome coronavirus 2 (SARS-CoV-2) Omicron infection in people with human immunodeficiency virus (HIV; PWH) with vaccine-induced or hybrid immunity. We assessed the incidence of Omicron infection in 209 AGE_h_IV coronavirus disease 2019 substudy participants with well-controlled HIV on antiretroviral therapy and 280 comparable controls, who had received at least the primary vaccination series.

**Methods:**

From September 2020 onward, participants were assessed every 6 months for the incidence of SARS-CoV-2 infection, per SARS-CoV-2 nucleocapsid antibody assay or self-reported positive antigen or polymerase chain reaction test. Between 1 January and 31 October 2022, the cumulative incidence of Omicron infection and associated risk factors were estimated using a conditional risk-set Cox proportional hazards model.

**Results:**

The cumulative incidence of a first Omicron infection was 58.3% by 31 October 2022, not significantly different between groups. HIV status was not independently associated with acquiring Omicron infection. Former and current smoking, as well as an increased predicted anti-spike immunoglobulin G titer were significantly associated with a lower risk of Omicron infection. The majority of infections were symptomatic, but none required hospitalization.

**Conclusions:**

People with well-controlled HIV and controls in our cohort experienced a similarly high proportion of Omicron infections. More booster vaccinations significantly reduced the risk of infection.

**Clinical Trial Registration.** NCT01466582

## INTRODUCTION

During the course of the coronavirus disease 2019 (COVID-19) pandemic severe acute respiratory syndrome coronavirus 2 (SARS-CoV-2) has evolved, and several dominant virus variants have emerged. From January 2022 onward, SARS-CoV-2 subvariants of the Omicron lineage (B.1.1.529) emerged in the Netherlands, with subvariants Omicron BA.1, BA.2, and BA.5 being dominant in 2022 [1]. Mutations in the spike (S) protein of the Omicron lineage appear to cause milder symptoms but are associated with increased transmissibility, partly because of resistance to host and/or vaccine-induced immune responses [2]. Previous general population studies have shown that neutralizing antibody titers against Omicron variants following primary vaccination are significantly lower than titers against ancestral SARS-CoV-2 [3–5]. Hence, despite of high SARS-CoV-2 vaccine uptake in the Netherlands (86.2% of Dutch adults had completed their primary vaccination as of January 2022 [6]), the number of confirmed infections increased rapidly since the emergence of the Omicron lineage [7].

A systematic review assessing the effectiveness of vaccination among the general population, either in those with prior SARS-CoV-2 non-Omicron infection (ie, hybrid immunity) or those without prior infection (ie, vaccine-induced immunity only), demonstrated that hybrid immunity provided greater and more sustained protection against the acquisition of an Omicron infection [8]. Moreover, having received additional booster vaccinations, compared with just the primary vaccination series, provided increased protection—both in people with [8] and those without [9] prior non-Omicron infection. Nonetheless, the effectiveness of hybrid immunity against Omicron infection ranged between only 41.8% at 12 months and 46.5% 6 months after primary vaccination and a first booster [8]. This effectiveness was significantly lower than that against SARS-CoV-2 infection with the Alpha or Delta variants [10].

Little is known about the incidence of Omicron infections in people with human immunodeficiency virus (HIV; PWH) with vaccine-induced or hybrid immunity. Given concerns about the immunogenicity of SARS-CoV-2 vaccines in PWH, despite their being virally suppressed by antiretroviral therapy (ART), many studies, including our own, have investigated (short-term) immune responses to SARS-CoV-2 vaccines in PWH [11, 12]. Before the emergence of the Omicron lineage, we reported robust and similar short-term humoral and cellular responses following the primary vaccination series in PWH and comparable controls, with higher responses in those with a prior infection [13]. Of note, in a subgroup of PWH and controls without prior infection in that study, neutralizing antibodies against replication-competent Omicron BA.1 could not be detected in any of the participants.

Although booster vaccinations seem to enhance the (short-term) neutralizing antibody response against Omicron variants [14, 15], it remains unclear whether such vaccine-induced responses and/or hybrid immunity from prior non-Omicron infections provide protection against Omicron infection in PWH. Thus, our aim was to assess the incidence and the determinants of Omicron infections in AGE_h_IV COVID-19 substudy participants with well-controlled HIV and comparable controls.

## METHODS

### Study Design and Participant Selection

Data were used from the AGE_h_IV COVID-19 substudy, the details of which have been described elsewhere [16]. In short, the AGE_h_IV Cohort Study is a prospective cohort study assessing the prevalence and incidence of aging-associated comorbid conditions and their risk factors in PWH and controls without HIV, aged ≥45 years at time of enrollment between 2010 and 2012 [17]. In September 2020, a COVID-19 substudy was initiated, including 567 AGE_h_IV Cohort participants in active follow-up and residing in the Netherlands, with five 6-monthly substudy visits between September 2020 and October 2022 (Supplementary Figure 1). During each of these visits, blood samples were obtained to assess SARS-CoV-2 immune responses, and participants completed a standardized study questionnaire.

For the current analysis, AGE_h_IV COVID-19 substudy participants were selected with ≥1 blood sample collected between 1 January 2022 (ie, the moment at which the Omicron lineage became dominant in the Netherlands) and 31 October 2022 (ie, the last possible date for participation in the final substudy visit). The analysis was limited to participants who had completed at least the primary vaccination series with any of the European Medicines Agency–approved SARS-CoV-2 vaccines used in the Netherlands by 1 January 2022: BNT162b2 (Pfizer-BioNTech), mRNA-1273 (Moderna), ChAdOx1 (AstraZeneca), Ad26.COV2.S (Janssen) and NVX-CoV237 (Novavax).

### Patient Consent Statement

Written informed consent was obtained from all participants. The study was approved by the ethics committee of the Amsterdam University Medical Centers, location Academic Medical Center, and is registered at www.clinicaltrials.gov (NCT01466582).

### Data Collection

#### Participant Characteristics

Data regarding date of birth, sex at birth, and ethnic origin had been obtained at time of enrollment into the parent AGE_h_IV Cohort Study. Data on other characteristics were obtained during the last available parent study visit before 1 January 2022 and included data on the number of prevalent comorbid conditions, body mass index, current CD4 and CD8 cell counts, last HIV test result for controls, and plasma HIV-1 RNA for PWH. PWH with HIV-1 RNA levels of <50 copies/mL or with transient viral blips of <200 copies/mL were considered virally suppressed.

#### Questionnaire Data

During each AGE_h_IV COVID-19 substudy visit, participants were asked to complete a standardized questionnaire, which assessed whether participants might have experienced any COVID-19–related symptoms and/or had been tested for SARS-CoV-2 infection. Furthermore, questions were included on substance use, sexual behavior, the use of ART in PWH, and HIV preexposure prophylaxis (PrEP) in controls. Finally, information was collected about SARS-CoV-2 vaccination status, vaccination dates, and vaccine types. Participants received their vaccinations at public health facilities, according to Dutch government guidelines [18].

#### Definition of SARS-CoV-2 Infection

At each substudy visit, SARS-CoV-2 nucleocapsid (N) antibody levels were measured using the semiquantitative INgezim COVID-19 double recognition assay (Eurofins Ingenasa), which captures the combined immunoglobulin (Ig) A, IgM, and IgG antibody response to the SARS-CoV-2 N protein (sensitivity, 100%; specificity, 98.2% [19]). Since SARS-CoV-2 vaccination involves immunity against only the SARS-CoV-2 S protein, an increase in N antibody levels results only from natural infection.

In participants with consecutively positive N antibody test results (eg, a positive N antibody result in both September 2021 and March 2022), we assessed whether these were indicative of a prior infection or reinfection. Evidence of reinfection (irrespective of the SARS-CoV-2 variant) can be revealed by measuring increases in N antibody levels. Participants who experience a SARS-CoV-2 infection have a rapid increase in N antibody levels, which subsequently decrease in the following months. An increase in N antibody levels (ie, ≥1.40-fold rise) between 2 consecutive blood draws within a time interval of <400 days is considered evidence of reinfection [20]. Further details and illustrative examples are provided in Supplementary Text 1 and Supplementary Table 1.

Given that sequencing results from nasopharyngeal swab samples were not accessible, the exact SARS-CoV-2 variant remained unknown. However, based on national epidemiological data [1], we assumed that all documented SARS-CoV-2 infections after 1 January 2022 involved the Omicron variant. Participants were considered to have a *definite* Omicron infection, either if they reported a positive antigen or polymerase chain reaction (PCR) test (nasal or nasopharyngeal swab) result between 1 January and 31 October 2022, or if the moment of SARS-CoV-2 N antibody seroconversion or ≥1.4-fold rise in N antibody levels occurred between March/April 2022 and September/October 2022. Participants in whom the moment of SARS-CoV-2 N antibody seroconversion or a ≥1.4-fold rise in N antibody levels occurred between September/October 2021 and March/April 2022, in the absence of an antigen or PCR test, were considered to have a *presumptive* Omicron infection.

In reporting the results of this study, those with an Omicron infection included all participants with either definite or presumptive Omicron infection, unless otherwise specified. A prior non-Omicron infection was defined by a self-reported positive antigen or PCR test result before 1 January 2022, or documented N antibody seroconversion between 1 September 2020 and 31 October 2021.

#### SARS-CoV-2 Humoral and Cellular Immune Responses

In addition, at each substudy visit, SARS-CoV-2 T-cell responses by IFN-γ–release assay, anti-S IgG production by memory B cells on polyclonal stimulation, and anti-S serum IgG titers by custom Luminex immunoassay were measured. Details have been described elsewhere [13] and are summarized in Supplementary Text 2.

### Statistical Analysis

Baseline was defined as 1 January 2022. Individual follow-up continued until the date of the last available SARS-CoV-2 N antibody measurement. Baseline characteristics were compared between PWH and controls using Pearson χ^2^, Fisher exact, or Wilcoxon rank sum tests, as appropriate.

The cumulative incidence of a first Omicron infection was estimated during 2 discrete time intervals (ie, from 1 January to 30 April 2022 [the end of the fourth substudy visit] and from 1 May to 31 October 2022 [the end of the last substudy visit]) and compared between PWH and controls using a log-rank test. Secondly, the cumulative incidence was compared between participants with a prior non-Omicron infection (ie, those with hybrid immunity) and those without (ie, those with vaccine-induced immunity only). In a sensitivity analysis, this comparison was limited to those considered having a definite Omicron infection.

As Omicron infections could occur several times during follow-up, rates of infection were modeled in a repeat-event analysis. Follow-up was divided into intervals of infection: follow-up began on 1 January 2022 and ended at the time of a first Omicron infection. Additional periods of follow-up were incorporated, beginning after the date of a first Omicron infection and ending at the date of a subsequent infection, until the last recorded infection. For individuals without evidence of an Omicron infection, follow-up continued until the last available SARS-CoV-2 N antibody measurement.

Hazard ratios comparing the incidence of an Omicron infection across levels of risk factors, along with 95% confidence intervals, were estimated using a conditional risk-set Cox proportional hazards model, allowing inclusion of follow-up time from multiple infections in a single participant. The following factors were assessed in the model: demographic and general clinical (HIV status, age, sex, ethnic origin, body mass index, and number of comorbid conditions); laboratory results (baseline CD4 and CD8 cell counts and CD4/CD8 ratio); HIV-specific variables (nadir CD4 cell count, years since HIV diagnosis, and years since start of first ART); behavioral (current alcohol and recreational drug use, smoking behavior [never/former/current] and history [pack-years], number of sexual contacts, and number of household contacts); SARS-CoV-2– and vaccine-related factors (prior non-Omicron infection, number of SARS-CoV-2 booster vaccinations received, and type of SARS-CoV-2 vaccine doses), and SARS-CoV-2 humoral and cellular immune responses (last known value of anti-S IgG titer, anti-S IgG production by memory B cells and T-cell IFN-γ release before Omicron infection in participants with an Omicron infection, and last known value in participants without an Omicron infection).

The final model was built using a backward stepwise selection procedure, including all variables associated with a *P* value <.20 in univariable analyses while removing those with a *P* value ≥.05 and forcing HIV status into the model. Given the strong relationship between the received number of SARS-CoV-2 boosters and SARS-CoV-2 anti-S IgG titers, predicted titers were estimated from a linear regression model with the received number of SARS-CoV-2 boosters as a covariate. These predicted values were used in the final proportional hazards model. Interactions between each significant variable in the final multivariable model and HIV status were tested in separate models. Finally, in participants with an Omicron infection, demographic, behavioral, and SARS-CoV-2– and vaccine-related characteristics, as well as clinical course of the infection (ie, asymptomatic or symptomatic and hospitalization status) were compared between PWH and controls.

## RESULTS

### Study Population

**Figure 1.**
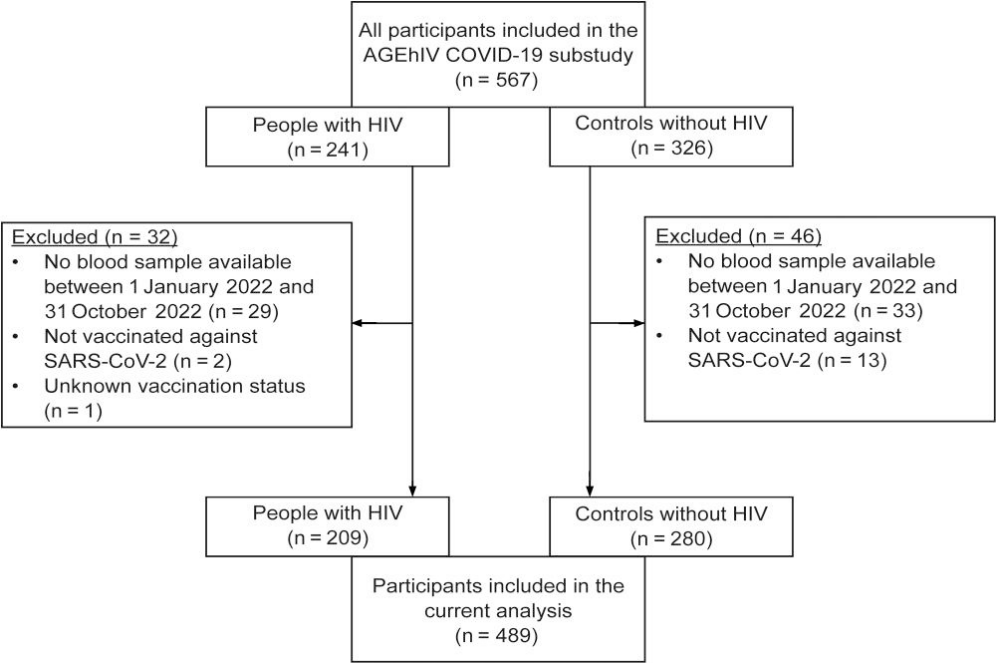
Inclusion of AGE_h_IV coronavirus disease 2019 (COVID-19) substudy participants in the current analysis. Abbreviations: HIV, human immunodeficiency virus; SARS-CoV-2, severe acute respiratory syndrome coronavirus 2.

Of the total 567 participants enrolled in the AGE_h_IV COVID-19 substudy, 78 were excluded for the following reasons: no blood sample available between January 1 and 31 October 2022 (n = 62), not vaccinated against SARS-CoV-2 (n = 15), or information on vaccination status missing (n = 1) (Figure 1). This resulted in 489 participants (209 PWH and 280 controls) being included in our analysis, whose characteristics are displayed in Table 1. The majority were male (88.6%), of Caucasian origin (96.1%), with a median age (interquartile range) of 61.9 (58.5–67.8) years. PWH were more often male and had more comorbid conditions. The median time since HIV diagnosis was 23.3 years, and the median nadir CD4 cell count was 190/µL. All PWH were receiving ART, and 99.5% were virally suppressed (<50 copies/mL, n = 206; blips 50–200 copies/mL, n = 2; 533 copies/mL, n = 1). The current median CD4 cell count in PWH was 670/µL, and ≥500/µL in 78.0%.

**Table 1.** Characteristics of the 489 Included AGE_h_IV Coronavirus Disease 2019 Substudy Participants, by HIV Status

Characteristic	Participants, No. (%)^a^		*P* Value
	PWH (n = 209)	Controls (n = 280)	
Age, median (IQR), y^b^	63.4 (59.1–68.3)	61.3 (58.1–67.2)	.058^c^
Age group, y^b^			
<60	68 (32.5)	111 (39.6)	.42^d^
60–64	58 (27.8)	73 (26.1)	
65–69	40 (19.1)	44 (15.7)	
≥70	43 (20.6)	52 (18.6)	
Male sex at birth	194 (92.8)	239 (85.4)	.01^d^
Ethnic origin			
Caucasian	200 (95.7)	270 (96.4)	.10^e^
African	8 (3.8)	4 (1.4)	
Asian	1 (.5)	6 (2.2)	
Educational level^f^			
Lower education (primary or secondary)	94/207 (45.4)	99/274 (36.1)	.04^e^
Higher vocational or university education	106/207 (51.2)	170/274 (62.0)	
Other	7/207 (3.4)	5/274 (1.8)	
BMI, median (IQR)^g^	24.7 (23.2–27.3)	25.0 (23.2–27.6)	.34^c^
BMI category^g^			
Underweight (<18.5)	3 (1.4)	0 (.0)	.23^e^
Normal weight (18.5–24.9)	108 (51.7)	137 (48.9)	
Overweight (25.0–29.9)	75 (35.9)	111 (39.6)	
Obese (≥30.0)	23 (11.0)	32 (11.4)	
No. of comorbid conditions^h,i^			
0	30 (14.4)	87 (31.1)	<.001^d^
1–2	121 (57.9)	160 (57.1)	
3–6	58 (27.8)	33 (11.8)	
Smoking behavior^h,j,k^			
Never smoker	65/193 (33.7)	95/265 (35.9)	.86^d^
Former smoker	95/193 (49.2)	124/265 (46.8)	
Current smoker	33/193 (17.1)	46/265 (17.4)	
Smoking history, pack-years^h,j,l^			
0	65/188 (34.6)	95/261 (36.4)	.94^d^
1–6	42/188 (22.3)	59/261 (22.6)	
7–19	33/188 (17.6)	47/261 (18.0)	
≥20	48/188 (25.5)	60/261 (23.0)	
Current alcohol use^h,j,k^	157/193 (81.4)	219/265 (82.6)	.72^d^
Current recreational drug use^h,j,k^	45/193 (23.3)	77/265 (29.1)	.18^d^
No. of household members^h,j,k^			
1 (Living alone)	88/193 (45.6)	131/265 (49.4)	.59^d^
2	94/193 (48.7)	116/265 (43.8)	
≥3	11/193 (5.7)	18/265 (6.8)	
No. of sexual contacts in last 6 mo^h,j,k^			
0–1	135/193 (70.0)	149/265 (56.2)	.01^d^
2–4	29/193 (15.0)	61/265 (23.0)	
≥5	29/193 (15.0)	55/265 (20.8)	
Current CD4 cell count, cells/µL^h^			
Median (IQR)	670 (520–840)	810 (630–1030)	<.001^c^
<350	14 (6.7)	4 (1.4)	<.001^e^
350–499	32 (15.3)	25 (8.9)	
500–749	82 (39.2)	83 (29.6)	
≥750	81 (38.8)	168 (60.0)	
Current CD8 cell count, cells/µL^h^			
Median (IQR)	690 (480–940)	410 (300–600)	<.001^c^
<350	28 (13.4)	105 (37.5)	<.001^e^
350–499	31 (14.8)	74 (26.4)	
500–749	58 (27.8)	49 (17.5)	
≥750	92 (44.0)	52 (18.6)	
Current CD4/CD8 ratio^h^			
Median (IQR)	.93 (.70–1.42)	1.97 (1.32–2.57)	<.001^c^
<0.50	15 (7.2)	0 (.0)	<.001^e^
0.50–0.99	97 (46.4)	27 (9.6)	
≥1.0	97 (46.4)	253 (90.4)	
Time since HIV diagnosis, median (IQR), y^b^	23.3 (17.4–28.5)	NA	…
Time since first starting ART, median (IQR), y^b^	20.9 (14.1–25.2)	NA	…
CD4 cell count nadir, median (IQR), cells/µL	190 (80–260)	NA	…
Undetectable HIV-1 viral load^h^	208 (99.5)	NA	…
Prior non-Omicron infection	34 (16.3)	41 (14.6)	.62^d^
Total no. of SARS-CoV-2 vaccine doses			
1	2 (1.0)	1 (.4)	.07^e^
2	17 (8.1)	31 (11.1)	
3	80 (38.3)	133 (47.5)	
4	88 (42.1)	97 (34.6)	
5	22 (10.5)	18 (6.4)	
SARS-CoV-2 vaccinations received			
Primary series only	17 (8.1)	26 (9.3)	.10^d^
Primary series + 1 booster	79 (37.8)	132 (47.1)	
Primary series + 2 boosters	90 (43.1)	103 (36.8)	
Primary series + 3 boosters	23 (11.0)	19 (6.8)	
SARS-CoV-2 vaccine type for primary vaccination series			
BNT162b2	132 (63.2)	182 (65.0)	.33^e^
mRNA-1273	8 (3.8)	9 (3.2)	
ChAdOx1	64 (30.6)	74 (26.4)	
Ad26.COV2.S	3 (1.4)	8 (2.9)	
ChAdOx1 + BNT162b2	2 (1.0)	2 (.7)	
Unknown	0 (.0)	5 (1.8)	
Time between last dose of primary series and 1st booster, median (IQR), d^m^	197 (181–220)	192 (179–209)	.03^c^
SARS-CoV-2 vaccine type for 1st booster			
BNT162b2	87 (41.6)	134 (47.9)	.45^e^
mRNA-1273	102 (48.8)	116 (41.4)	
Unknown	3 (1.4)	4 (1.5)	
No booster	17 (8.1)	26 (9.3)	
Time between 1st and 2nd boosters, median (IQR), d^n^	116 (99–199)	116 (96–188)	.57^c^
SARS-CoV-2 vaccine type for 2nd booster			
BNT162b2	26 (12.5)	29 (10.4)	.11^e^
mRNA-1273	82 (39.2)	90 (32.1)	
Unknown	5 (2.4)	3 (1.1)	
No 2nd booster	96 (45.9)	158 (56.4)	
Time between 2nd and 3rd boosters, median (IQR), d^o^	190 (173–202)	190 (110–207)	.47^c^
SARS-CoV-2 vaccine type for 3rd booster			
BNT162b2	5 (2.4)	2 (.7)	.26^e^
mRNA-1273	15 (7.2)	13 (4.6)	
Unknown	3 (1.4)	4 (1.4)	
No 3rd booster	186 (89.0)	261 (93.2)	

Abbreviations: ART, antiretroviral therapy; BMI, body mass index; HIV, human immunodeficiency virus; IQR, interquartile range; mRNA, messenger RNA; NA, not applicable; PWH, people with HIV.

^a^Data represent no. (%) of participants unless otherwise specified.

^b^At baseline (defined as 1 January 2022).

^c^Calculated using Wilcoxon rank sum test.

^d^Calculated using Pearson χ^2^ test.

^e^Calculated using Fisher exact test.

^f^Missing in 2 of 209 PWH and 6 of 280 controls.

^g^BMI calculated as weight in kilograms divided by height in meters squared.

^h^Last available data before baseline.

^i^In the last 6 months.

^j^Total comorbidity count includes cardiovascular disease, cancer, chronic kidney disease, diabetes mellitus, hypertension, obesity, and chronic obstructive pulmonary disease.

^k^Missing in 16 of 209 PWH and 15 of 280 controls.

^l^Missing in 21 of 209 PWH and 19 of 280 controls.

^m^In 191 of 192 PWH and 250 of 254 controls with a first booster vaccination after the primary series.

^n^In 112 of 113 PWH and 121 of 122 controls with 2 boosters.

^o^In 23 of 23 PWH and 19 of 19 controls with 3 boosters.

Most participants had received BNT162b2 (64.2%) or ChAdOx1 (28.2%) for their primary vaccination series. The majority (n = 446 [91.2%]) had subsequently received ≥1 BNT162b2 or mRNA-1273 booster vaccinations; 211 participants had received 1 booster, 193 had received 2, and 42 had received 3. The total number of SARS-CoV-2 vaccinations, vaccine types, and the median time between vaccinations did not differ between PWH and controls (Table 1).

At baseline, 75 participants (15.3%; 34 [16.3%] with and 41 [14.6%] without HIV] had evidence of prior non-Omicron infection. Only 1 participant (with suppressed HIV and a current CD4 cell count of 1050/µL) had had 2 documented non-Omicron infections (ie, including a non-Omicron reinfection) during study follow-up.

### Incidence of Omicron Infections

Among all participants, 277 (56.6%) acquired a first Omicron infection during follow-up (PWH, n = 110 of 209 [52.6%]; controls, n = 167 of 280 [59.6%]; *P* = .12). In 47 of the 277 (17.0%) (PWH, n = 19 of 110 [17.3%]; controls, n = 28 of 167 [16.8%]; *P* = .91), there was documented evidence of a prior non-Omicron infection.

Of the 277 infections, 133 (48.0%) were identified by a self-reported positive antigen or PCR test result between 1 January and 31 October 2022 and were thus considered definite Omicron infections, either with (n = 93 of 133) or without (n = 40 of 133) N antibody seroconversion or a ≥1.4-fold rise in N antibody levels (details in Supplementary Text 3). The remaining 144 of 277 Omicron infections (52.0%) were identified by a documented N antibody seroconversion or a ≥1.4-fold rise in N antibody levels either between September/October 2021 and March/April 2022 (presumptive Omicron infections, n = 57 of 144 [39.6%]) or between March/April and September/October 2022 (definite Omicron infections, n = 87 of 144 [60.4%]). These percentages did not differ significantly between PWH and controls (Supplementary Table 2).

**Figure 2.**
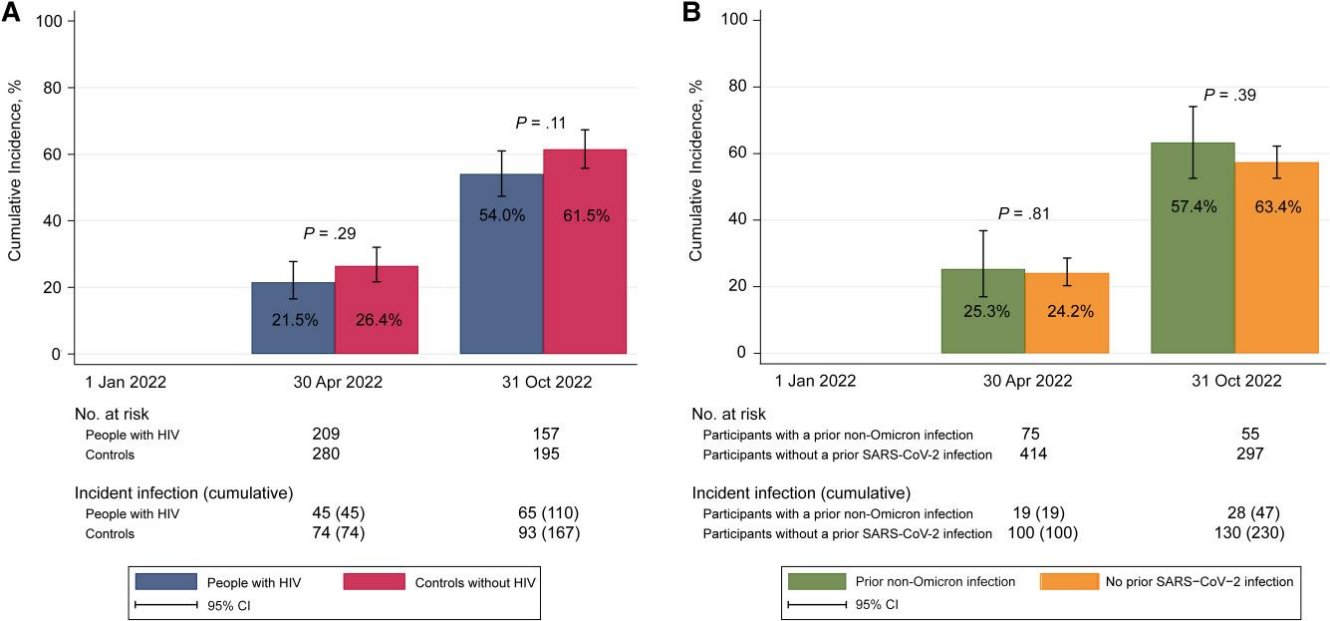
Cumulative incidence of a first severe acute respiratory syndrome coronavirus 2 (SARS-CoV-2) Omicron infection from 1 January until 31 October 2022 among included AGE_h_IV coronavirus disease 2019 substudy participants. Cumulative incidences of a first Omicron infection were compared between people with human immunodeficiency virus (HIV) and controls (*A*) and between participants with and those without a prior non-Omicron infection (*B*). Follow-up started on 1 January 2022 and continued until the date of the last SARS-CoV-2 nucleocapsid antibody measurement. The numbers of participants at risk and with incident infection (and the cumulative number of infections) at the end of each time interval are shown. The 7 second Omicron infections (Omicron reinfections) are not displayed in these plots. *P* values are based on log-rank test.

During follow-up, 7 of the 277 participants with a first Omicron infection demonstrated evidence of a second Omicron infection, including 1 participant with HIV and 6 controls (2 of the 6 also had a prior non-Omicron infection). The cumulative incidence of a first Omicron infection in the cohort overall was 24.3% by 30 April 2022 and increased to 58.3% by 31 October 2022. The cumulative incidence did not differ significantly between PWH and controls (Figure 2*A*), nor between participants with or without a prior non-Omicron infection (Figure 2*B*). In the sensitivity analysis which excluded participants with a presumptive Omicron infection (n = 57), the cumulative incidence by 31 October 2022 was likewise not significantly different between PWH and controls, albeit numerically lower in PWH (47.8% and 56.6%, respectively; *P* = .054).

### Factors Associated With Acquiring Omicron Infection

HIV status was not independently associated with acquiring Omicron infection in either univariable (Supplementary Table 3) or multivariable (Table 2) analyses. In the final multivariable model, only former and current smoking and a higher predicted anti-S IgG titer (as a function of the number of SARS-CoV-2 booster vaccinations received) were significantly associated with a lower risk of Omicron infection. Neither of these had a significant interaction with HIV status. In PWH, current CD4 and CD8 cell count, CD4 cell count nadir, years since HIV diagnosis, and years since ART initiation were not associated with acquiring Omicron infection.

**Table 2.** Factors Associated With Severe Acute Respiratory Syndrome Coronavirus 2 Omicron Infection Acquired Between 1 January and 31 October 2022 Among 489 Participants in the AGE_h_IV Coronavirus Disease 2019 Substudy

Factor	Conditional Risk-Set Cox Proportion Hazards Model			
	Univariable		Multivariable	
	HR (95% CI)	*P* Value	aHR (95% CI)	*P* Value
HIV status				
Negative	Reference	.11	Reference	.28
Positive	.86 (.71–1.04)		.89 (.73–1.09)	
Smoking behavior^a,b^				
Never smoker	Reference	<.001	Reference	<.001
Former smoker	.80 (.65–.99)		.81 (.66–1.00)	
Current smoker	.52 (.37–.73)		.51 (.36–.71)	
Predicted anti-spike IgG titer by Luminex (per 1-log-unit increase)^c,d^	.46 (.33–.65)	<.001	.45 (.32–.63)	<.001

Abbreviations: aHR, adjusted hazard ratio; CI, confidence interval; HIV, human immunodeficiency virus; HR, hazard ratio; IgG, immunoglobulin G.

^a^Last available data before severe acute respiratory syndrome coronavirus 2 (SARS-CoV-2) nuleocapsid antibody test.

^b^In the last 6 months.

^c^Last known value before Omicron infection in participants with an Omicron infection or last known value in those without an Omicron infection.

^d^Predictions were obtained from a linear regression model whose parameter estimates are as follows: difference in mean anti-spike IgG titer relative to receipt of primary SARS-CoV-2 vaccination series alone, +0.25 (95% CI, .088–.41) for primary series plus 1 booster; +0.35 (.19–.51) for primary series plus 2 boosters; and +0.35 (.14–.55) for primary series plus 3 boosters (*P* <. 001).

### Clinical Course of Omicron Infection in PWH and Controls

None of the 277 participants with Omicron infection was admitted to the hospital. In participants with available information on symptoms (n = 260), 15.0% did not experience any symptoms in the period between the last study visit and the Omicron infection. In those with experienced symptoms, the most frequently reported were rhinorrhea, fatigue, cough, and headache. These findings did not differ between PWH and controls. Demographic, behavioral, and SARS-CoV-2 vaccine–related characteristics were also similar between PWH and controls with an Omicron infection (Table 3).

**Table 3.** Characteristics of 277 AGE_h_IV Coronavirus Disease 2019 Substudy Participants With Severe Acute Respiratory Syndrome Coronavirus 2 Omicron Infection, by HIV Status

Characteristic	Participants, No. (%)^a^		*P* Value
	PWH (n = 110)	Controls (n = 167)	
Omicron infection^b,c^			
Asymptomatic	17/101 (16.8)	22/159 (13.8)	.51^d^
Symptomatic	84/101 (83.2)	137/159 (86.2)	
Symptoms^c^			
Fever	27/101 (26.7)	37/159 (23.3)	.69^d^
Chills	37/101 (36.6)	45/159 (28.3)	.30^d^
Rhinorrhea	59/101 (58.4)	100/159 (62.9)	.77^d^
Ear pain	6/101 (5.9)	17/159 (10.7)	.41^e^
Cough	54/101 (53.5)	92/159 (57.9)	.77^d^
Phlegm	37/101 (36.6)	56/159 (35.2)	.68^d^
Sore throat	37/101 (36.6)	70/159 (44.0)	.30^d^
Shortness of breath	23/101 (22.8)	45/159 (28.3)	.55^d^
Loss of smell	11/101 (10.9)	13/159 (8.2)	.48^d^
Loss of taste	7/101 (6.9)	10/159 (6.3)	.91^e^
Fatigue	58/101 (57.4)	86/159 (54.1)	.78^d^
Muscle ache	39/101 (38.6)	54/159 (34.0)	.60^d^
Headache	47/101 (46.5)	70/159 (44.0)	.89^d^
Confusion	3/101 (3.0)	10/159 (6.3)	.55^e^
Nausea	12/101 (11.9)	16/159 (10.1)	.90^d^
Vomiting	6/101 (5.9)	5/159 (3.1)	.50^e^
Abdominal pain	10/101 (9.9)	14/159 (8.8)	.86^d^
Diarrhea	25/101 (24.8)	23/159 (14.5)	.11^d^
Chest pain	12/101 (11.9)	14/159 (8.8)	.72^d^
Other	4/101 (4.0)	8/159 (5.0)	.41^e^
Hospital admission due to COVID-19^c^	0 (.0)	0 (.0)	>.99^e^
Age, median (IQR), y^f^	62.1 (58.5–66.7)	61.4 (57.8–67.4)	.46^g^
Age group, y			
<60	40 (36.4)	69 (41.3)	.68^d^
60–64	30 (27.3)	39 (23.3)	
65–69	21 (19.1)	26 (15.6)	
≥70	19 (17.3)	33 (19.8)	
Male sex at birth	102 (92.7)	139 (82.2)	.03^e^
Ethnic origin			
Caucasian	105 (95.5)	160 (95.8)	.04^d^
African	5 (4.5)	2 (1.2)	
Asian	0 (.0)	5 (3.0)	
BMI, median (IQR)^h,i^	24.7 (23.2–26.8)	25.4 (23.2–28.2)	.27^g^
BMI category^i^			
Underweight (<18.5)	2 (1.8)	0 (.0)	.33^e^
Normal weight (18.5–24.9)	56 (50.9)	79 (47.3)	
Overweight (25.0–29.9)	37 (33.6)	66 (39.5)	
Obese (≥30.0)	15 (13.7)	22 (13.2)	
Total no. of comorbid conditions^h,j^			
0	13 (11.8)	49 (29.3)	<.001^d^
1–2	65 (59.1)	96 (57.5)	
3–6	32 (29.1)	22 (13.2)	
Current smoking^b,h,k^	11/101 (10.9)	20/159 (12.6)	.68^d^
Current alcohol use^b,h,k^	85/101 (84.2)	132/159 (83.0)	.81^d^
Current recreational drug use^b,h,k^	29/101 (28.7)	48/159 (30.2)	.84^d^
No. of household members^h,j,l^			
1 (living alone)	43/100 (43.0)	79/159 (49.7)	.35^e^
2	53/100 (53.0)	70/159 (44.0)	
≥3	4/100 (4.0)	10/159 (6.3)	
No. of sexual contacts in last 6 mo^b,h,k^			
0–1	65/101 (64.4)	88/159 (55.4)	.08^d^
2–4	19/101 (18.8)	25/159 (15.7)	
≥5	17/101 (16.8)	46/159 (28.9)	
Prior non-Omicron infection	19 (17.3)	28 (16.8)	.91^d^
SARS-CoV-2 vaccinations received			
Primary vaccination series only	12 (10.9)	16 (9.6)	.97^e^
Primary vaccination series + 1 booster	52 (47.3)	83 (49.7)	
Primary vaccination series + 2 boosters	39 (35.4)	57 (34.1)	
Primary vaccination series + 3 boosters	6 (6.4)	11 (6.6)	
SARS-CoV-2 vaccine type primary vaccination series			
BNT162b2	73 (66.4)	115 (68.9)	.74^e^
mRNA-1273	3 (2.7)	4 (2.4)	
ChAdOx1	33 (30.0)	41 (24.6)	
Ad26.COV2.S	1 (.9)	4 (2.4)	
ChAdOx1 + BNT162b2	0 (.0)	1 (.6)	
Unknown	0 (.0)	2 (1.2)	
SARS-CoV-2 vaccine type 1st booster			
BNT162b2	50 (45.5)	78 (46.7)	.98^e^
mRNA-1273	47 (42.7)	72 (43.1)	
Unknown	1 (.9)	1 (.6)	
No booster	12 (10.9)	16 (9.6)	
SARS-CoV-2 vaccine type 2nd booster			
BNT162b2	6 (5.5)	16 (9.6)	.57^e^
mRNA-1273	38 (34.5)	50 (29.9)	
Unknown	2 (1.8)	2 (1.2)	
No 2nd booster	64 (58.2)	99 (59.3)	
SARS-CoV-2 vaccine type 3rd booster			
BNT162b2	1 (.9)	1 (.6)	>.99^e^
mRNA-1273	5 (4.6)	8 (4.8)	
Unknown	1 (.9)	2 (1.2)	
No third booster	103 (93.6)	156 (93.4)	

Abbreviations: BMI, body mass index; COVID-19, coronavirus disease 2019; IQR, interquartile range; PWH, people with human immunodeficiency virus; SARS-CoV-2, severe acute respiratory syndrome coronavirus 2.

^a^Data represent no. (%) of participants unless otherwise specified.

^b^Missing in 9 of 110 PWH and 8 of 167 controls.

^c^In the period since last study visit and Omicron infection.

^d^Pearson χ^2^ test.

^e^Fisher exact test.

^f^At baseline (defined as 1 January 2022).

^g^Wilcoxon rank sum test.

^h^Last available data before date of Omicron infection.

^i^BMI calculated as weight in kilograms divided by height in meters squared.

^j^Total comorbidity count includes cardiovascular disease, cancer, chronic kidney disease, diabetes mellitus, hypertension, obesity and chronic obstructive pulmonary disease .

^k^In the last 6 months.

^l^Missing in 10 of 110 PWH and 8 of 167 controls.

## DISCUSSION

After the SARS-CoV-2 Omicron variant had become dominant in the Netherlands in early 2022, the cumulative incidence of infection rapidly increased to >50% by the end of October that year, in both PWH and controls in our study who had all completed at least their primary vaccination series against SARS-CoV-2. The acquisition of Omicron infection in both groups was similar for those with hybrid or only vaccine-induced immunity. An increased predicted anti-S IgG titer and current or former smoking were the only factors independently associated with a reduced risk of Omicron infection. The majority of infections (about 85% in both groups) were likely symptomatic, but none resulted in hospitalization.

HIV status was not independently associated with the risk of Omicron infection, and neither were HIV-specific parameters in PWH, similar to what we had reported for the same cohort during the first 14 months of the pandemic when most participants were unvaccinated [16]. However, the power to assess the association between CD4 cell count or HIV viral load and incident Omicron infection was limited, with 78% of PWH in our study having a CD4 cell count ≥500/µL and 95% an undetectable viral load. Nonetheless, our findings are consistent with those of other studies conducted before the emergence of Omicron, which also did not find associations between HIV status, CD4 cell count, or HIV viral load and the risk of acquiring a SARS-CoV-2 infection in PWH [21–23]. Thus far, only 1 study from China in 890 PWH and 1364 adults without HIV, of whom >90% had been vaccinated with ≥1 dose of an inactivated SARS-CoV-2 vaccine, found that the prevalence of Omicron infection was lower among PWH. CD4 cell count was not associated with the risk of Omicron infection in PWH in this study [24].

The predicted anti-S IgG titer—estimated from a linear regression model with a significant association between receipt of ≥1 booster vaccinations and anti-S IgG titers—was associated with a lower risk of Omicron infection. Several studies have already demonstrated that SARS-CoV-2 booster vaccinations enhance humoral immune responses in PWH [15, 25–28], but to our knowledge our study is the first to show that these responses are also associated with a lower risk of Omicron infection in PWH.

The significant association of current and former smoking with a lower risk of Omicron infection is a surprising observation. Interestingly, current smoking was also associated with a lower risk of SARS-CoV-2 infection in our previous study, albeit significantly only in univariable analysis (unadjusted hazard ratio, 0.42 [95% confidence interval, .18–.99]) [16]. Such a counterintuitive protective effect of smoking has also been reported by a number of pre-Omicron studies in the general population, with some of those in addition reporting smoking to be associated with an increased risk of severe COVID-19 outcomes [29–31]. In the absence of a plausible biological mechanism by which smoking would offer protection against acquiring SARS-CoV-2 infection, our observation and those of others should be interpreted with great caution, as they may be confounded by unmeasured social determinants of health.

Approximately 15% of Omicron infections in both PWH and controls were asymptomatic, which is lower than the pooled percentage of 25.5% [32] to 32.4% [33] reported in 2 systematic reviews among individuals positive for the Omicron variant (by antigen or PCR test). Of note, heterogeneity among studies was high in both reviews. A possible explanation for the lower percentage in our study may be the high prevalence of comorbid conditions (76.1% of participants had ≥1 chronic comorbid condition) and former and current smokers in our cohort, with participants possibly reporting symptoms related rather to these comorbid conditions and/or smoking than to actual symptoms of Omicron infection. Furthermore, we asked participants to report symptoms experienced over a 6-month period rather than just in the weeks surrounding confirmed infection. This is a limitation of our data, which warrants caution when interpreting data on symptoms associated with an Omicron infection. Finally, our participants' median age of 62 years may also have contributed to a higher likelihood of developing symptoms [34].

Almost half of Omicron infections in our cohort were identified by a self-reported positive antigen or PCR test result. Of those, 30.1% did not demonstrate N antibody seroconversion or a ≥1.4-fold rise in N antibody level. Recent studies have indeed shown the rate of N antibody seroconversion and the level of N antibody response following SARS-CoV-2 infection to be significantly lower in SARS-CoV-2–vaccinated than in unvaccinated individuals [35, 36]. Omicron infections may therefore also have been missed among those without a self-reported positive antigen or PCR test result and those without N antibody seroconversion or a ≥1.4-fold rise in N antibody levels. Given the similar percentage of PWH and controls without N antibody seroconversion or a ≥1.4-fold rise in N antibody levels among those with a self-reported positive antigen or PCR test result, we expect our main conclusion to likely remain largely unchanged.

To our knowledge, ours is the first longitudinal study comparing the incidence of Omicron infection between vaccinated PWH and controls. A key strength is the well-characterized highly comparable population of PWH and controls with respect to demographic and lifestyle characteristics. Furthermore, detailed information was available on SARS-CoV-2 vaccinations (doses and types) and prior non-Omicron infection in all participants and on detailed HIV-specific characteristics in PWH.

Our study also has limitations. First, participants in whom the moment of SARS-CoV-2 N antibody seroconversion or a ≥1.4-fold rise in N antibody levels occurred between September/October 2021 and March/April 2022, in the absence of an antigen or PCR test, may in fact have been infected before 1 January 2022, and thus infected with a non-Omicron variant. Although a sensitivity analysis in which these participants were excluded demonstrated a somewhat lower cumulative incidence of Omicron infection by 31 October 2022 (52.8% vs 58.3% in the primary analysis), consistently there was no significant difference between PWH and controls. Second, we were not able to control for differences in the specificity and sensitivity of antigen versus PCR testing as our questionnaire did not distinguish between antigen and PCR testing. Furthermore, it was unknown whether participants received an adapted SARS-CoV-2 vaccine specifically targeting Omicron BA subvariants. These adapted vaccines, however, were only introduced in the Netherlands by mid-September 2022—that is, near the end of our data collection—and are thus unlikely to have affected our results. Finally, our findings may not apply equally to parts of the world where PWH do not have access to or have less optimal responses to ART.

In conclusion, people with well-controlled HIV on ART and controls in our cohort, who had all received a variety of primary SARS-CoV-2 vaccines and ≥1 mRNA booster vaccination, experienced similarly high proportions of incident Omicron infection. Having received multiple booster vaccinations significantly reduced the risk of infection. None of the incident Omicron infections resulted in hospitalization, which suggests substantial sustained protection against severe COVID-19. Although these findings may not be generalizable to all PWH, they are reassuring for the fortunately sizable and increasing global population of PWH with a good virological and immunological response to ART.

## Supplementary Data

Supplementary materials are available at *Open Forum Infectious Diseases* online. Consisting of data provided by the authors to benefit the reader, the posted materials are not copyedited and are the sole responsibility of the authors, so questions or comments should be addressed to the corresponding author.

## Supplementary Material

ofae380_Supplementary_Data
