# Supplementary material for: Similar Limited Protection Against Severe Acute Respiratory Syndrome Coronavirus 2 Omicron Infection in Vaccinated Individuals With HIV and Comparable Controls
Source: Open Forum Infect Dis. 2024 Jul 8;11(7):ofae380. doi: 10.1093/ofid/ofae380 (PMC11273239; doi:10.1093/ofid/ofae380)
Supplement: ofae380_Supplementary_Data [file ofae380_supplementary_data.docx]

**SUPPLEMENTARY DATA**

**TO MANUSCRIPT**: Similar limited protection against SARS-CoV-2 Omicron infection in vaccinated individuals with HIV and comparable controls

**AUTHORS**

Myrthe L. Verburgh, Anders Boyd, Maarten F. Schim van der Loeff, Margreet Bakker, Ferdinand W.N.M. Wit, Marc van der Valk, Marloes Grobben, Lisa van Pul, Khadija Tejjani, Jacqueline van Rijswijk, Marit J. van Gils, Neeltje A. Kootstra, Lia van der Hoek, Peter Reiss; on behalf of the AGE_h_IV Cohort Study*

* Study group members are listed in the Acknowledgments

**CONTENT**

Figure S1 …………………………………………………………………………….. page 2

Text S1 …………………………………………………………………………….. page 3

Table S1 …………………………………………………………………………….. page 4

Text S2 …………………………………………………………………………….. page 5

Text S3 …………………………………………………………………………….. page 6

Table S2 …………………………………………………………………………….. page 7

Table S3 …………………………………………………………………………….. page 8

**Supplementary Figure S1. Design of the AGE_h_IV Cohort Study and COVID-19 substudy.**


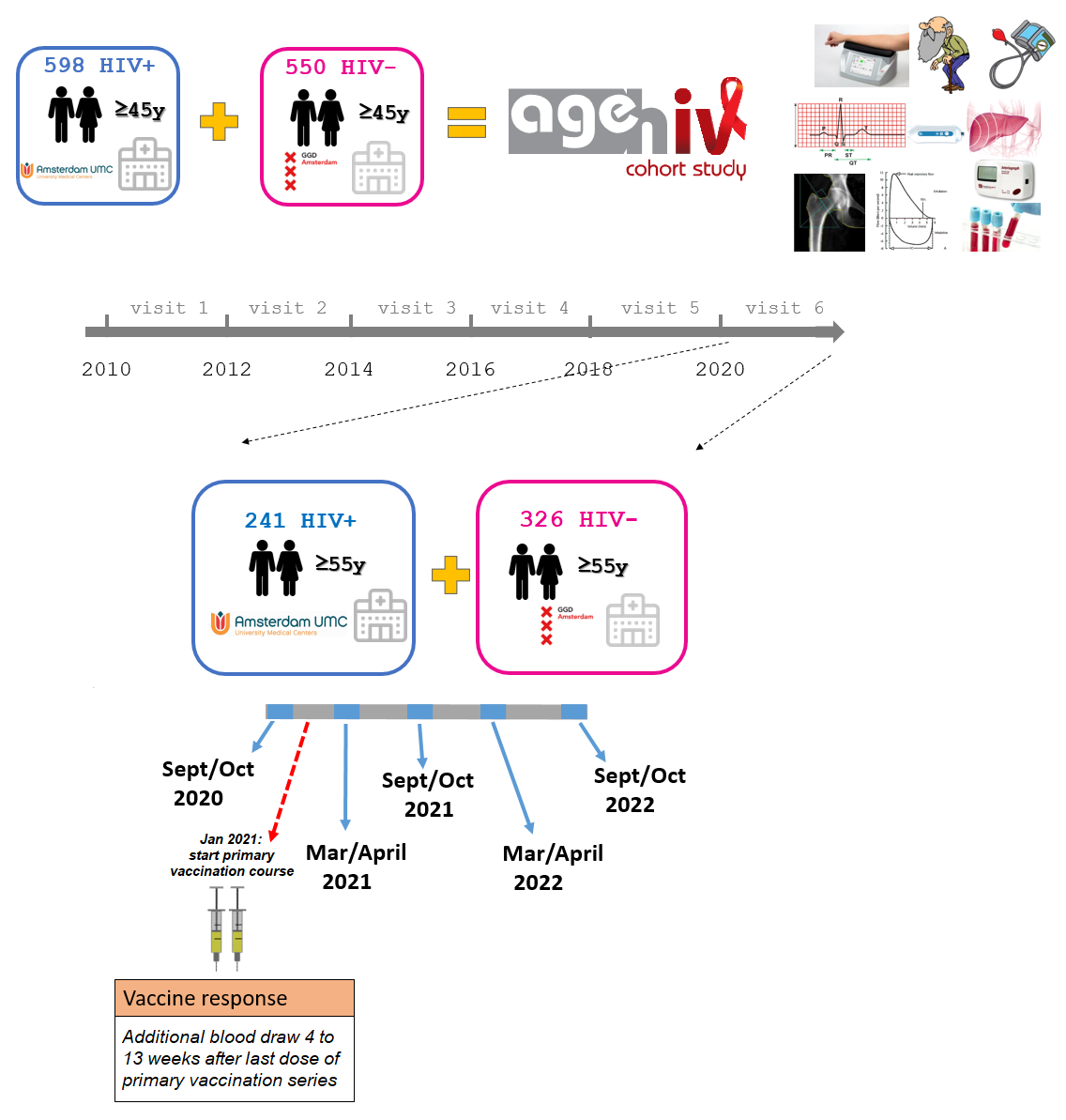


In the prospective observational AGE_h_IV Cohort Study, 598 people with HIV were enrolled at the outpatient HIV-clinic of the Amsterdam University Medical Centers (Amsterdam UMC), location AMC, and 550 controls without HIV from the sexual health clinic and the Amsterdam Cohort Studies on HIV/AIDS at the Public Health Service Amsterdam between 2010 and 2012. Every two years, patients undergo screening for age-associated comorbidities.

In September 2020, a COVID-19 substudy was initiated, including 567 AGE_h_IV Cohort participants in active follow-up and residing in the Netherlands, and encompassed five six-monthly study visits between September 2020 and October 2022. In addition to these five substudy visits, participants were invited for an additional blood draw 4 to 13 weeks after completing the primary vaccination series. The Dutch national COVID-19 vaccination program started in January 2021.

**Supplementary Text S1. Two examples to illustrate the assessment of SARS-CoV-2 (re)infections based on IgA/IgM/IgG nucleocapsid-antibody levels via the semiquantitative INgezim COVID-19 double recognition assay.**

Nucleocapsid (N)-antibody levels were expressed as a ratio of the sample to positive control for each sample, calculated as follows: [(OD) sample − OD blank)/(OD positive control − OD blank)) × 10, where OD represents optical density. A sample–positive control ratio ≥6 was considered a positive SARS-CoV-2 N antibody response, in accordance with the manufacturer’s instructions, and was used as evidence of N-antibody seroconversion and defining a first SARS-CoV-2 infection.

In participants with consecutively positive N-antibody tests, we assessed whether the N-antibody levels of second or later measurements were indicative of a prior SARS-CoV-2 infection or rather of a reinfection. A renewed increase in N-antibody levels (fold rise ≥1.40) between two consecutive blood draws (within a time interval shorter than 400 days) is considered as evidence of a reinfection. **In a previous study led by one of our co-authors (L. van der Hoek)[1], seasonal coronavirus infections were determined by measuring fold changes in optical density (OD) in an N-antibody recognition ELISA when tested on serum from consecutive visits. To determine the cut-off, the natural fluctuation in antibody signals when no infection occurred was determined by testing measles virus antibodies every six months in individuals that did not have a measles infection during follow-up. The fold changes in measles virus antibody OD ranged between 0.85 and 1.28. Subsequently, the authors examined the distribution of OD fold change for seasonal coronaviruses during a total 206 person-years of follow-up with six-monthly sampling. When looking at the distribution of OD-fold changes during long periods of time, it can be assumed that outlying values of this distribution (i.e., 1.5 x IQR above the 75-percentile or below the 25-percentile) are indicative of new infections.[1] The authors observed that fold changes >= 1.40 were outlying values and indeed corresponded to higher neutralization titers against live virus and increases in serum neutralization titers.[1]**

**In the current study, based on the above mentioned findings, we extrapolated these results to SARS-CoV-2, and considered an increase of ≥1.40 fold in N-antibodies indicative of (re-)infection with SARS-CoV-2.**

As shown in Table S1, participant Example001 had a first positive N-antibody test on March 15, 2021, indicative of a first *non-Omicron* infection. The N-antibody tests at the four consecutive time points all remained positive; but the change in N-antibody level from the previous visit only exceeded 1.40-fold on September 21, 2022. This represents a reinfection, or, for the purposes of this analysis, a first *Omicron* infection.

Participant Example002 had a first positive N-antibody test on September 28, 2021, indicative of a first *non-Omicron* infection. The N-antibody tests at the two consecutive time points remained positive; however, the change in N-antibody level from the previous visit did not exceed 1.40-fold. Hence, there is no evidence of a reinfection based on these N-antibody tests.

**References**:

1. Edridge AWD, Kaczorowska J, Hoste ACR, et al. Seasonal coronavirus protective immunity is short-lasting. Nat Med **2020**; 26:1691-3.

**Supplementary Table S1. Nucleocapsid-antibody test results and their interpretation in two participants of the AGE_h_IV COVID-19 substudy.**

| **Participant number** | **Date of N-antibody test** | **Result** | **N-antibody level** | **Fold change in N-antibody level compared to previous test** | **Interpretation** |
| --- | --- | --- | --- | --- | --- |
| Example001 | 24 Sept 2020 | negative | 0.59 |  |  |
| Example001 | 15 Mar 2021 | positive | 41.65 | 71.20 | First SARS-CoV-2 infection (non-Omicron variant) |
| Example001 | 7 Jul 2021 | positive | 27.48 | 0.66 | No reinfection based on N-antibody levels |
| Example001 | 29 Sept 2021 | positive | 20.48 | 0.75 | No reinfection based on N-antibody levels |
| Example001 | 29 Mar 2022 | positive | 15.30 | 0.75 | No reinfection based on N-antibody levels |
| Example001 | 21 Sept 2022 | positive | 59.56 | 3.89 | SARS-CoV-2 reinfection (Omicron variant) |
|  | | | | | |
| Example002 | 15 Sept 2020 | negative | 0.62 | - |  |
| Example002 | 12 Apr 2021 | negative | 1.40 | - |  |
| Example002 | 28 Sept 2021 | positive | 23.87 | 17.05 | First SARS-CoV-2 infection (non-Omicron variant) |
| Example002 | 1 Mar 2022 | positive | 7.67 | 0.32 | No reinfection based on N-antibody levels |
| Example002 | 22 Sept 2022 | positive | 6.71 | 0.87 | No reinfection based on N-antibody levels |

Abbreviations: N-antibody, nucleocapsid-antibody.

**Supplementary Text S2. Detailed methods of SARS-CoV-2 humoral and cellular immune response measurements.**

SARS-CoV-2 humoral immune responses

SARS-CoV-2 anti-spike (S) IgG titers against the Wuhan strain were measured in serum using an in-house custom Luminex immunoassay. In short, SARS-CoV-2 S-proteins were covalently coupled to Luminex MagPlex beads. Beads were incubated with 1:100,000-diluted serum overnight at 4°C. The next day, the beads were washed followed by a 2-hour incubation at room temperature with phycoerythrin (PE)-labeled goat anti-human IgG (Southern Biotech, Birmingham, AL, USA). Beads were washed, and readout was performed on a Magpix machine (Luminex, Austin, TX, USA). The resulting values are expressed as the median fluorescence intensity (MFI) of at least 50 beads per protein. Beads with tetanus toxoid and respiratory syncytial virus F protein (RSV-F) as a positive control and beads with no protein as a negative control were also included in every well. Positive- and negative-control sera were included on every plate as well as a titration of convalescent COVID-19 patient sera to monitor assay performance.

SARS-CoV-2 anti-S IgG production by memory B cells was determined in peripheral blood mononuclear cells (PBMCs) upon polyclonal stimulation. A total of 1 × 10^6^ PBMCs were stimulated for 5 days with requisimod (R848; 1 μg/mL) (InvivoGen, San Diego, CA, USA) and IL-2 (10 U/mL) (Chiron Benelux, Amsterdam, The Netherlands) and cultured in RPMI 1640 culture medium supplemented with penicillin, streptomycin, and 10% heat-inactivated fetal bovine serum (FBS). Culture supernatant was harvested for determination of SARS-CoV-2 anti-S IgG production by enzyme-linked immunosorbent assay (ELISA) using SARS-CoV-2 S-protein coating and anti-human IgG conjugated with horseradish peroxidase (Southern Biotech, Birmingham, AL, USA). Pooled serum of convalescent COVID-19 patients was included in each plate as a positive control. SARS-CoV-2 IgG levels in the culture medium are given relative to the positive control. The lower limit of detection was 0.5 relative units (RU) per 10^6^ PBMCs, and the lower limit of quantification was 1 RU per 10^6^ PBMCs.

SARS-CoV-2 cellular immune responses

SARS-CoV-2-specific T-cell responses were measured using an IFN-γ release assay. A total of 0.5 × 10^6^ PBMCs isolated from obtained blood were stimulated with a SARS-CoV-2 nucleocapsid (N)- and S-peptide pool (JPT Peptide Technologies, Berlin, Germany) or cultured in medium alone as a control. After 24 h, culture supernatants were harvested and IFN-γ released by the cells was determined by human IFN-γ DuoSet ELISA (R&D Systems, Minneapolis, MN, USA). SARS-CoV-2-induced IFN-γ release (picograms per milliliter) was determined by subtraction of background IFN-γ production (medium alone) from that in the SARS-CoV-2 peptide-stimulated culture.

**Supplementary Text S3. Details on 40 participants with a self-reported positive antigen- or PCR-test, indicative of an Omicron infection, without nucleocapsid-antibody seroconversion or ≥1.4 fold rise in N-antibody levels, in case of earlier seroconversion.**

Of 133 participants with a self-reported positive antigen- or PCR-test, indicative of an Omicron infection, 40 (15 PWH, 25 controls) did not exhibit nucleocapsid (N)-antibody seroconversion or ≥1.4 fold rise in N-antibody levels. N-antibody levels were expressed as a ratio of the sample to positive control for each sample, calculated as follows: [(OD) sample − OD blank)/(OD positive control − OD blank)) × 10, where OD represents optical density. A sample–positive control ratio ≥6 was considered a positive SARS-CoV-2 N-antibody response, in accordance with the manufacturer’s instructions. The fold rise in N-antibody levels was determined only in case of two consecutive positive N-antibody measurements in an individual.

Of those 40 participants, 13 did show a response on N-antibody levels, but those levels did not reach the threshold of 6 (median N-antibody level 5.0 [IQR 3.8-5.5]). One participant had a positive N-antibody measurement, however compared to his/her previous positive N-antibody measurement, it did not reach the ≥1.4 fold rise in N-antibody levels.

In 9 of 40 participants, the N-antibody measurement was performed ≥6 months after the date of the self-reported positive antigen- or PCR-test. Hence, N-antibody levels may have waned over time to below the threshold of 6 or did not reach the ≥1.4 fold rise in N-antibody levels in those individuals.

Moreover, in 7 of 40 participants, the N-antibody measurement was performed within 2 weeks after the date of the self-reported positive antigen- or PCR-test. In those individuals, N-antibody levels may not have yet risen enough to reach the threshold of 6 or the ≥1.4 fold rise in N-antibody levels.

The remaining 10 participants had N-antibody levels below 3, hence no indication of N-antibody level rises, despite having a self-reported positive antigen- or PCR-test.

When comparing PWH and controls, as shown in Table Text S3 below, controls were more likely to have no indication of N-antibody level rises.

**Table Text S3. Participants with a self-reported positive antigen- or PCR-test without nucleocapsid-antibody seroconversion or ≥1.4 fold rise in N-antibody levels, stratified by HIV-status.**

|  | **People with HIV**  **(n = 15)** | **Controls**  **(n=25)** |
| --- | --- | --- |
| **N-antibody levels between 3 and 6** | 5 (33.3%) | 8 (32.0%) |
| **No ≥1.4 fold rise in N-antibody levels** | 0 (0.0%) | 1 (4.0%) |
| **N-antibody measurement ≥6 months after date of self-reported positive antigen- or PCR-test** | 6 (40.0%) | 3 (12.0%) |
| **N-antibody measurement <2 weeks after date of self-reported positive antigen- or PCR-test** | 2 (13.3%) | 5 (20.0%) |
| **No indication of N-antibody level rises (N-antibody level <3)** | 2 (13.3%) | 8 (32.0%) |

**Supplementary Table S2. Identified SARS-CoV-2 Omicron infections between January 1, 2022 and October 31, 2022 among participants of the AGE_h_IV COVID-19 substudy, stratified by HIV-status.**

|  | **People with HIV with an Omicron infection**  **(n = 110)** | **Controls with an Omicron infection**  **(n = 167)** | ***P*** |
| --- | --- | --- | --- |
| **Omicron infection identified by**  A) Self-reported positive antigen- or PCR-test (test date between Jan 1 and Oct 31, 2022)  B) Documented N-antibody seroconversion or ≥1.4 fold rise in N-antibody levels between Sept/Oct 2021 and Mar/Apr 2022  C) Documented N-antibody seroconversion or ≥1.4 fold rise in N-antibody levels between Mar/Apr 2022 and Sept/Oct 2022 | 48 (43.6%)  25 (22.7%)  37 (33.6%) | 85 (50.9%)  32 (19.2%)  50 (29.9%) | .49 |

The classification per category is hierarchical, because identification of Omicron infection by means of a positive antigen- or PCR- test was regarded as the strongest evidence. For example: if a participant met the classification for both A) and B), this participant is classified under A). Abbreviations: P, p-value; PCR, polymerase chain reaction; N-antibody, nucleocapsid-antibody.

**Supplementary Table S3. Factors associated with** **SARS-CoV-2 Omicron infection acquired between January 1, 2022 and October 31, 2022 among 489 participants of the AGE_h_IV COVID-19 substudy.**

|  | Univariable analysis | |
| --- | --- | --- |
|  | **HR (95% CI)** | ***P*** |
| HIV status  HIV-negative  HIV-positive | REF  0.86 (0.71 – 1.04) | .11 |
| Age ^A^  <60 years  60-64 years  65-69 years  ≥70 years | 1.14 (0.88 – 1.46)  0.98 (0.73 – 1.31)  1.08 (0.78 – 1.49)  REF | .60 |
| Sex at birth  Male  Female | REF  1.14 (0.87 – 1.49) | .33 |
| Ethnic origin  Caucasian  African  Asian | REF  0.95 (0.55 – 1.66)  1.20 (0.67 – 2.13) | .82 |
| BMI ^B^  Underweight (<18.5 kg/m^2^)  Normal weight (18.5-24.9)  Overweight (25.0-29.9)  Obese (≥30.0 kg/m^2^) | 1.00 (0.32 – 3.19)  REF  1.11 (0.90 – 1.38)  1.23 (0.92 – 1.65) | .50 |
| Total comorbidities ^B, C^  0 comorbidities  1-2 comorbidities  3-6 comorbidities | REF  1.05 (0.83 – 1.33)  1.17 (0.87 – 1.57) | .58 |
| Current alcohol consumption ^B, D^  No  Yes | REF  1.10 (0.83 – 1.44) | .51 |
| Current recreational drug use ^B, D^  No  Yes | REF  1.11 (0.89 – 1.38) | .37 |
| Smoking behaviour ^B, D^  Never smoker  Former smoker  Current smoker | REF  0.80 (0.65 – 0.99)  0.52 (0.37 – 0.73) | <.001 |
| Smoking pack years ^B, D^  0 pack years  1-6 pack years  7-19 pack years  ≥20 pack years | REF  0.68 (0.52 – 0.91)  0.88 (0.68 – 1.14)  0.65 (0.49 – 0.85) | .0048 |
| Number of household members ^B, D^  One person (living alone)  Two persons  3 or more persons | REF  1.02 (0.83 – 1.27)  0.92 (0.56 – 1.50) | .90 |
| Number of sexual contacts in the last six months ^B, D^  0 – 1 partners  2 – 4 partners  5 or more partners | REF  1.04 (0.76 – 1.41)  1.20 (0.94 – 1.54) | .36 |
| Current CD4 cell count ^B^  <350 cells/mm^3^  350-499 cells/mm^3^  500-749 cells/mm^3^  ≥750 cells/mm^3^ | 1.07 (0.61 – 1.87)  0.89 (0.64 – 1.23)  0.81 (0.65 – 1.02)  REF | .30 |
| Current CD8 cell count ^B^  <350 cells/mm^3^  350-499 cells/mm^3^  500-749 cells/mm^3^  ≥750 cells/mm^3^ | REF  0.77 (0.57 – 1.04)  0.81 (0.62 – 1.06)  0.97 (0.76 – 1.24) | .20 |
| Current CD4/8 ratio ^B^  < 0.50  0.50 – 0.99  ≥ 1.0 | 1.16 (0.62 – 2.20)  0.84 (0.66 – 1.07)  REF | .32 |
| Time since HIV diagnosis ^A, E^  (per year increase) | 0.99 (0.96 – 1.01) | .18 |
| Time since ART initiation ^A, E^  (per year increase) | 0.98 (0.96 – 1.00) | .099 |
| CD4 nadir ^E^  (per 100 cells/mm^3^ decrease) | 0.95 (0.85 – 1.06) | .34 |
| Prior non-Omicron infection  No  Yes | REF  1.20 (0.93 – 1.54) | .16 |
| SARS-CoV-2 vaccinations received  Primary vaccination series only  Primary vaccination series + one booster  Primary vaccination series + two boosters  Primary vaccination series + three boosters | REF  0.85 (0.63 – 1.13)  0.59 (0.44 – 0.81)  0.48 (0.30 – 0.77) | <.001 |
| SARS-CoV-2 vaccine type primary vaccination series  BNT162b2  mRNA-1273  ChAdOx1  Ad26.COV2.S  ChAdOx1 + BNT162b2  Unknown | REF  0.76 (0.38 – 1.53)  0.92 (0.74 – 1.14)  0.74 (0.36 – 1.51)  0.36 (0.06 – 2.01)  3.53 (0.91 – 13.76) | .27 |
| Anti-spike IgG titer ^F^  (per one log-unit increase) | 0.41 (0.33 – 0.50) | <.001 |
| Anti-spike IgG production by memory B-cells ^F^  (per one log-unit increase) | 0.69 (0.58 – 0.82) | <.001 |
| T-cell IFNy release ^F^  (per one log-unit increase) | 0.92 (0.87 – 0.97) | .002 |

Values represent unadjusted hazard ratios (HR) with 95% confidence interval. A. At baseline (defined as January 1, 2022). B. Last available data prior to the SARS-CoV-2 N-antibody test. C. Total comorbidity count includes cardiovascular disease, cancer, chronic kidney disease, diabetes mellitus, hypertension, obesity and chronic obstructive pulmonary disease (COPD). D. In the last six months. E. Only in people with HIV (PWH) (n=209). F. Last known value prior to Omicron infection in participants with an Omicron infection or last known value in participants without an Omicron infection. Abbreviations: ART, antiretroviral therapy; BMI, body mass index; CI, confidence interval; HR, hazard ratio; P, p-value; NA, not applicable; REF, reference group
